# *In silico* prospection of microorganisms to produce polyhydroxyalkanoate from whey: *Caulobacter segnis* DSM 29236 as a suitable industrial strain

**DOI:** 10.1111/1751-7915.13371

**Published:** 2019-01-31

**Authors:** Daniel Bustamante, Silvia Segarra, Marta Tortajada, Daniel Ramón, Carlos del Cerro, María Auxiliadora Prieto, José Ramón Iglesias, Antonia Rojas

**Affiliations:** ^1^ Biopolis, S.L. Parque Científico Universidad de Valencia edf. 2 C/Catedrático Agustín Escardino, 9 46980 Paterna Valencia Spain; ^2^ Microbial and Plant Biotechnology Department Centro de Investigaciones Biológicas Madrid Spain; ^3^ Corporación Alimentaria Peñasanta (CAPSA) Polígono Industrial 0, 33199 Granda, Asturias Spain; ^4^ Present address: National Renewable Energy Laboratory (NREL) 15013 Denver West Parkway Golden CO 80401 USA

## Abstract

Polyhydroxyalkanoates (PHAs) are polyesters of microbial origin that can be synthesized by prokaryotes from noble sugars or lipids and from complex renewable substrates. They are an attractive alternative to conventional plastics because they are biodegradable and can be produced from renewable resources, such as the surplus of whey from dairy companies. After an *in silico* screening to search for ß‐galactosidase and PHA polymerase genes, several bacteria were identified as potential PHA producers from whey based on their ability to hydrolyse lactose. Among them, *Caulobacter segnis* DSM 29236 was selected as a suitable strain to develop a process for whey surplus valorization. This microorganism accumulated 31.5% of cell dry weight (CDW) of poly(3‐hydroxybutyrate) (PHB) with a titre of 1.5 g l^−1^ in batch assays. Moreover, the strain accumulated 37% of CDW of PHB and 9.3 g l^−1^ in fed‐batch mode of operation. This study reveals this species as a PHA producer and experimentally validates the *in silico* bioprospecting strategy for selecting microorganisms for waste re‐valorization.

## Introduction

The use of industrial secondary manufacturing streams as feedstocks is one of the main objectives of the bioeconomy. Therefore, processing methods are used in biorefineries to develop more sustainable and environmentally friendly technologies. Industrial biotechnology and the development of specialized microbial factories, including genetically modified organisms (GMOs), may offer alternative paths to conventional processes, and, consequently, proof of concept has been shown that many products traditionally produced from petroleum like plastic materials can now be synthesized using renewable resources. Alternative bio‐based plastics offering the same functionalities of traditional plastics with potentially lower environmental impacts are being developed, but at the moment represent a very small share of the market (Prieto, 2016). Increasing the uptake of alternatives that according to solid evidence are more sustainable can also help decrease our dependency on fossil fuels (http://ec.europa.eu/environment/circular-economy/pdf/plastics-strategy-annex.pdf). In this scenario, the current socioeconomic trend towards sustainable development models has promoted research into the generation of bioplastics such as polyhydroxyalkanoates (Braunegg *et al*., 2004; Dietrich *et al*., 2017; Koller *et al*., 2017).

Polyhydroxyalkanoates have a renewable origin, with similar properties to polyolefins. They are biodegradable and biocompatible. Moreover, their water and oxygen permeability is low, and therefore, they can be used in packaging (Verlinden *et al*., 2007; Koller, 2014). Their hydrolysis products (3‐R‐hydroxyalkanoic acids) are optically active molecules of high pharmaceutical interest (Chen and Wu, 2005). The properties of PHA depend on the length of the lateral chain: short‐chain‐length PHAs, such as poly(3‐hydroxybutyrate; PHB), have higher crystallinity, are thermoplastic and behave as polypropylene, whereas medium‐chain‐length PHAs behave like elastomers. They have a wide range of applications in medicine, pharmacy, veterinary and food packaging (Chen, 2009; Brigham and Sinskey, 2012; Dinjaski and Prieto, 2015; *Raza et al.,* 2018). The challenge facing this clean production technology lies in the ability to obtain bioplastics at a similar or lower cost than those from petrochemical origin. Some small‐ and middle‐sized companies produce PHA at small scale, for example P&G (USA), Biomer Inc. (Germany), Tianan Biologic (China) and PHB Industrial (Brazil; Jiang *et al*., 2016).

The price of these products is still higher than their conventional counterparts, although in recent years some companies report values as low as $1.50 kg^−1^ (Chanprateep, 2010). Lately, efforts have been made to improve the fermentation and extraction process required to produce them, as well as to isolate and develop more productive microbial strains that can use low‐cost substrates (Khanna and Srivastava, 2005; Mozejko‐Ciesielska and Kiewisz, 2016). In fact, raw material accounts for 30–40% of the total cost of PHB; therefore, using renewable raw materials is fundamental to obtain profitable and environmentally friendly processes. Raw materials may include waste material from other industrial processes (Castilho *et al*., 2009; Sathya *et al*., 2018), such as whey.

Whey is generated in cheese factories on separating the milk curd and is one of the most polluting materials generated by the food industry due to its high organic and mineral content. It is a product rich in protein, fat and lactose (Spalatelu, 2012; Fernández‐Gutiérrez *et al*., 2017). Studies have assessed the recovery of whey as a source of lactic acid, xanthan gum, lactitol and lactulose. In this respect, it is interesting to consider the production of bioplastics and derivatives thereof from whey (Mollea *et al*., 2013; Pescuma *et al*., 2015). Global production of whey is estimated at around 90 × 10^6^ ton per year and growing. With milk and cheese production rising globally each year by 2% and 3%, respectively, evidence suggests that the volume of whey produced will continue to increase in the coming years (Ryan and Walsh, 2016). Thus, advances in the field of biotechnology to develop sustainable methods of dealing with whey are necessary. In the same vein, the optimization of PHA production processes is an interesting approach for exploiting the potential of whey.

Polyhydroxyalkanoates are produced as carbon and energy reserves or reducing power storage materials in the presence of excess carbon, especially with limitation of other essential nutrients such as oxygen, nitrogen or phosphorus (Koller *et al*., 2008; Anjum *et al*., 2016). Some bacteria are able to use the lactose in whey to synthesize PHA. The prior art reports different types of PHA produced from whey or lactose by Gram‐negative bacteria (*Methylobacterium* sp., *Pseudomonas* sp. or *Thermus thermophilus*) and Gram‐positive bacteria, some lactic acid bacteria (genera *Lactobacillus* and *Lactococcus*) and *Bacillus megaterium* (Koller *et al*., 2012; Pais *et al*., 2014, 2016). Within the *Caulobacter* genus, the species *C. crescentum* has been described as producing PHA from glucose as carbon source (Qi and Rehm, 2001).

The development of genetic engineering techniques and intensive studying of metabolic potential of microorganisms has allowed designing genetically modified microorganisms (GMOs). They are applied in a variety of fields such as human health, agriculture, bioremediation and different types of industry (Cases and de Lorenzo, 2005; Jones *et al*., 2015). However, there is a strong social debate, mainly in the European Union, about the use of GMOs (Sankar and Cho, 2015). To select wild‐type microorganisms that transform residual streams, such as whey, into a product of interest in an efficient way is a challenging issue. The strategies can be (i) culture‐based approaches consisting in the isolation of microorganisms from residual sources and (ii) *in silico* prospection of wild‐type microorganisms that takes advantages of the large amount of data generated by genome and metagenome sequencing projects (Walsh *et al*., 2015; Zarins‐Tutt *et al*., 2016).

Since PHA production is a step forward in the processing of whey and has the potential to ecologically overcome fossil polymers as shown in life cycle studies (Koller *et al*., 2013; Narodoslawsky *et al*., 2015), the aim of this work was to find new bacterial species capable of producing PHA from whey with high yield, through an *in silico* prospection and subsequent experimental validation.

## Results and discussion

### *In silico* identification of PHA‐producing microorganisms from lactose

*In silico* bioprospecting was performed generating, in a first step, a list of organisms described in literature with the ability to produce PHA and reported β‐galactosidase activity (Fig. S1). Another group of microorganisms was listed based on BLAST searches using *E. coli* β‐galactosidase protein sequence (NP_414878) as prototype enzyme for hydrolysing lactose, towards bibliographically studied PHA producers with sequenced genomes. Subsequently, *E. coli* β‐galactosidase protein sequence and prototypic PHA polymerase protein sequences belonging to the four described types (Table 1; Pettinari *et al*., 2001; Rehm, 2003; Valappil *et al*., 2007; Tsuge *et al*., 2015) were used to perform BLAST searches in the UniProt database. Those organisms with positive hits in both searches were also selected. Finally, prototypic protein sequences were used to perform Tblastn searches against nucleotide sequences in the GeneBank database and organisms with both putative functions were selected. All the selected strains characteristics were manually revised, and data on culture conditions and biosafety level were retrieved for each case. Thus, 42 potential PHA‐producing strains with the ability to hydrolyse lactose were identified. We discarded from the list all those strains described as pathogenic or those requiring growth conditions difficult to reproduce on an industrial scale due to special requirements, such as vitamins or high temperatures (Table 2).

**Table 1:** Genes used for *in silico* searching potential strains for PHA production from whey

Gene	Organism	Accession number
β‐galactosidase (*lacZ*)	*Escherichia coli* K12	NP_414878
PHA polymerase Type I (*phaC*)	*Ralstonia eutropha*	WP_011615085
PHA polymerase Type II (*phaCI*)	*Pseudomonas putida* KT2440	WP_010955566
PHA polymerase Type II (*phaCII*)	*Pseudomonas putida* KT2440	WP_010955568
PHA polymerase Type III (*phaC*)	*Allochromatium vinosum*	WP_012969309
		WP_012969310
PHA polymerase Type IV (*phaC*)	*Bacillus megaterium*	WP_013055939
		WP_034653582

**Table 2:** Selected microorganisms from the literature and *in silico* search to study PHA production using lactose and whey

Species	Strain	Putative Polymerase	Polymerase Acc. Number	β‐Galactosidase Acc. Number	References
*Azohydromonas lata*	DSM 1123	I	WP_066338481	–	Baei *et al*. (2010)
*Amycolatopsis mediterranei*	DSM 43304	II	WP_013228250	WP_013226436	Verma *et al*. (2011)
*Asticcacaulis excentricus*	DSM 4724	I	WP_013480283	WP_013481046	Skerman *et al*. (1980)
				WP_013480849	
				WP_013478732	
*Azotobacter vinelandii*	DSM 2289	I	WP_012700949	–	Koller *et al*. (2007)
*Bacillus megaterium*	DSM 90	IV	WP_013055939	–	Chen *et al*. (1991)
			WP_034653582		
*Bacillus mycoides*	CECT 4128T	IV	WP_002011573	WP_033798304	Borah *et al*. (2002)
*Bacillus pseudomycoides*	CECT 7065T	IV	WP_003196066	WP_033799124	Zwick *et al*. (2012)
*Caulobacter segnis*	DSM 29236	I	WP_041538528	WP_013078956	Urakami *et al*. (1990)
*Hydrogenophaga pseudoflava*	DSM 1034	I	–	–	Koller *et al*. (2007)
*Novosphingobium aromaticivorans*	DSM 12444	I, IV	WP_041549985	WP_011446066	Takeuchi *et al*. (2001)
			WP_011906798		
*Oceanicola granulosus*	DSM 15982	I	WP_040614877	WP_007256669	Cho and Giovannoni (2004)
*Photobacterium angustum*	DSM 19184	II	WP_045149949	WP_045132314	Reichelt *et al*. (1976)
*Pseudomonas hydrogenovora*	DSM 1749	I	–	–	Koller *et al*. (2008)
*Rhizobium meliloti*	CECT 4114T	III	WP_027993898	WP_027991400	Tombolini *et al*. (1995)
*Sphingopyxis alaskensis*	DSM 13593	I	WP_041383587	WP_011541322	Lauro *et al*. (2009)
*Tolumonas auensis*	DSM 9187	I	WP_049759190	WP_015879948	Chertkov *et al*. (2011)
*Tsukamurella paurometabola*	CECT 3055 T	II	WP_013128530	WP_013125671	Munk *et al*. (2011)
*Vibrio coralliilyticus*	DSM 19607	I	WP_006961110	WP_039952109	Ushijima *et al*. (2014)
*Vibrio orientalis*	DSM 19136	I	WP_004411639	WP_004413027	Yang *et al*. (1983)
*Vibrio shilonii*	DSM 13774	I	WP_006072398	WP_006071939	Kushmaro *et al*. (2001)
				WP_006070128	
				WP_031493251	

### Characterization of the whey and evaluation of PHA production from lactose

First, the whey and permeate composition was determined. The term ‘whey’ refers to the liquid fraction of the milk that is separated from the curd during cheese production, whereas ‘permeate’ is produced when protein is removed from the whey as so‐called ‘retentate’ fraction. We analysed lactose content, lactic acid, protein, pH, etc., and the results are shown in Table 3. The results confirmed that it has the expected composition for whey (Spalatelu, 2012; Fernández‐Gutiérrez *et al*., 2017) which makes it a good source of nutrients for the growth of microorganisms and suitable to evaluate the production of PHA with the selected strains. Additionally, the composition (% wt) of the whey according to the elemental analysis was determined as follows: 30.05% carbon, 6.65% hydrogen, 1.90% nitrogen and 61.4% oxygen.

**Table 3:** Characterization of whey and permeate to be employed in the formulation of culture media for PHA production

	Whey	Permeate
[Lactose] g l^−1^	38 ± 1.5	56 ± 1.2
[Lactic acid] g l^−1^	0.8 ± 0.2	0.1 ± 0.05
[Citric acid] g l^−1^	0.7 ± 0.1	0.1 ± 0.02
[Protein] g l^−1^	6.7 ± 0.5	3.2 ± 0.5^a^
[Fat] g l^−1^	4.0 ± 0.3	0.1 ± 0.01
Ash (%)	0.5 ± 0.1	0.6 ± 0.06
Total Solids (%)	6.5 ± 0.4	5.5 ± 0.8
pH	6.6 ± 0.1	6.2 ± 0.1

**a**. Non‐protein nitrogen included in this value. May or may not contain true protein.

**Figure 1:**
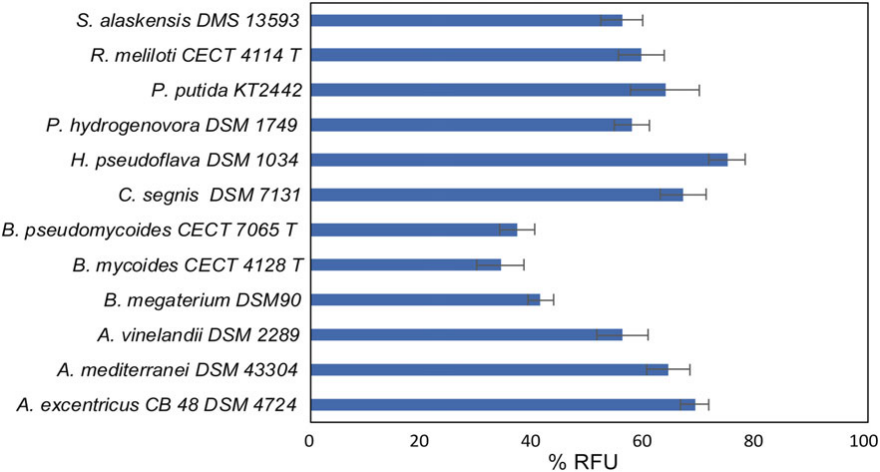
Bacterial strains selected from Nile red assays. Experiment was done in 300‐μl multiwell plates with the strains growing in each medium selected to test PHA accumulation by Nile red staining method (see Experimental procedures). *Escherichia coli* DH5α was used as negative control to PHA production, and *Pseudomonas putida* KT2442 was used to test Nile red staining as PHA producer (from glucose). Tests were performed in triplicate. Percentage of RFU was calculated considering the RFU measurement before and after adding Nile red to the samples and taking *E. coli* DH5α result as reference. % RFU = ∆RFU_S__train_‐∆RFU_*E*__*.coli*_/∆RFU_S__train_

It was necessary to develop a method to allow a rapid estimation of the PHA‐accumulating abilities of a large number of strains and whereby PHA‐producing bacteria could be quickly distinguished from non‐producing bacteria. The protocol developed is based on staining of the PHA with Nile red as prescreening until analysis confirmation by GC. Nile red binds to hydrophobic inclusions within the cell including the PHA. Fluorescence increases in correlation with an increase in the amount of accumulated polymer. This method compares the fluorescence of Nile red‐stained cells in an environment that does not produce PHA with the fluorescence in a medium that promotes PHA production (Spiekermann *et al*., 1999). *Pseudomonas putida* KT2442 was used to test the effectiveness of Nile red staining for being a good PHA producer and *Escherichia coli* DH5α (ATCC 11303) as a negative control for being non‐PHA‐producing strain (Solaiman, 2000; Prieto *et al*., 2007). The non‐production conditions were set with LB medium, whereas PHA accumulation conditions were tested using three different media: 0.1 N M63 medium with glucose or lactose as carbon sources to compare with diluted whey and permeate as culture medium (see Experimental procedures for details). Relative fluorescence unit (RFU) measurements were taken after 24 h of incubation because it was sufficient time to evaluate the growth and production of PHA considering the amount of nitrogen source available in the culture media selected for the trial. In addition, measuring the RFU at 48 h of incubation showed no improvement with any of the strains. The results of the screening based on Nile red staining are shown in Table S1. Seawater isolated strains such as *Oceanicola granulosus* DSM 15982, *Photobacterium angustum* DSM 19184, *Vibrio coralliilyticus* DSM 19607, *Vibrio orientalis* DSM 19136 and *Vibrio shilonii* DSM 13774 showed very slow growth in the culture media selected for the experiment. Most of the strains grew in LB, whey and/or permeate and not in the medium 0.1 N M63 due to more complex nutritional requirements. Some strains were able to accumulate PHA in LB medium or using the fatty acids and/or proteins of the whey as a carbon source. However, to continue with the selection of strains, the results obtained with whey were analysed separately, taking into account that using the crude substrate is the best option against any type of pretreatment. Therefore, the percentage of relative fluorescence units (%RFU) was calculated from the difference between the values of RFU before and after adding Nile red and taking as reference that value for *E. coli* DH5α in whey as negative control. Figure 1 shows the strains in which there was accumulation of PHA according to the test. *Pseudomonas putida* KT2442 does not consume lactose, but it accumulated PHA from other carbon sources contained in the whey, presumably from fatty acids. Strains with growth difficulties, with special growing conditions or with complex nutritional requirements, were discarded. This is the case of the marine strains or strains that need the addition of vitamins or complex carbon sources in the culture medium, such as *Azohydromonas lata* DSM 1123, *Novosphingobium aromaticivorans* DSM 12444 or *Tolumonas auensis* DSM 9187. In general, the more complex a culture medium is and the more extreme the growth conditions of a microorganism are, the greater the costs for scaling‐up the process (Koller, 2017).

Finally, five strains were selected for further testing and quantification of PHA production: *Amycolatopsis mediterranei* DSM 43304, *Caulobacter segnis* DSM 29236, *Pseudomonas hydrogenovora* DSM 1749 (today: *Paraburkholderia fungorum,* according to www.dsmz.de, Coenye *et al*., 2001), *Bacillus megaterium* DSM 90 and *Hydrogenophaga pseudoflava* DSM 1034. The following assays were performed in MM1 medium. In tests with diluted whey to lactose 12 g l^−1^, sugar consumption did not exceed 10% (Table S2). *Hydrogenophaga pseudoflava*,* P. hydrogenovora* and *B. megaterium* are well‐known PHA producers (Koller *et al*., 2008; Obruca *et al*., 2009). The production of PHA by *A. mediterranei* and *C. segnis* had not been described before. It was decided to continue with *C. segnis* DSM 29236 because of its novelty and because it appeared to be the producer with the highest capacity according to the preliminary results.

### Selection of optimal fermentation process conditions for PHA production

**Figure 2:**
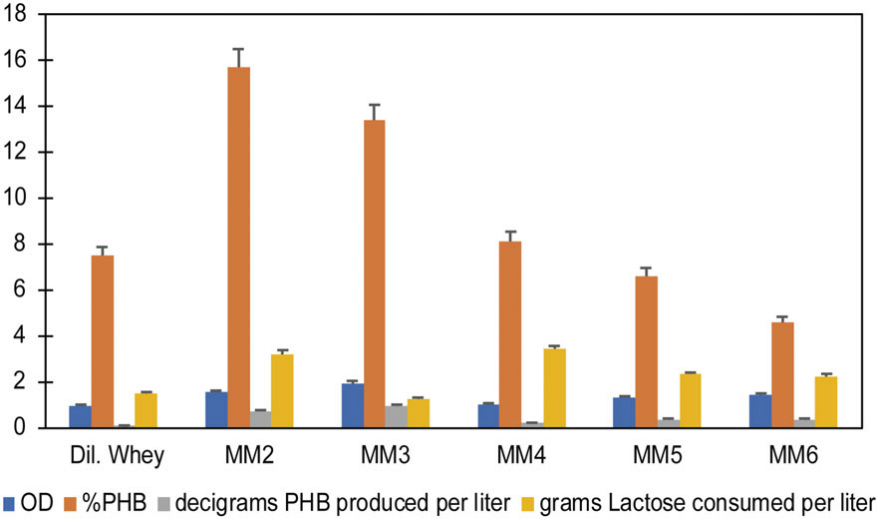
Optimization of PHB production from whey with *Caulobacter segnis* DSM 29236. Different media were used as follows: MM2, MM3, MM4, MM5 and MM6, with an initial lactose concentration of 12 g l^−1^. Assays were performed in flasks at 30°C, 150 rpm for 48 h (see Experimental procedures).

The PHA produced by *C. segnis* DSM 29236 was identified as poly‐3‐hydroxybutyrate (PHB; Fig. S2). The available sequence of the PHA polymerase found in the published *C. segnis* genome shows a class I poly(R)‐hydroxyalkanoic acid synthase (accession number WP_0415385280). For class I PHA polymerases, *Ralstonia eutropha* is considered as the model strain (Rehm, 2003). For *R. eutropha* and related species to *C. segnis* such as *Caulobacter crescentus*, PHA metabolism involves the synthesis of acetyl‐CoA from sugars and its subsequent conversion into PHB (Farinha, 2009; Buckley, 2013). Different culture media were used to optimize PHB production from whey with *C. segnis* DSM 29236: diluted whey, MM2, MM3, MM4, MM5 and MM6, with an initial lactose concentration of 12 g l^−1^. The highest PHB titre was obtained with medium MM3. The strain produced 0.09 g l^−1^ of PHB with a 13.4% accumulation of its cell dry weight (CDW; Fig. 2), but lactose was not totally consumed. This medium contains a minimum amount of nitrogen source and contains phosphates, unlike the MM4 medium.

**Figure 3:**
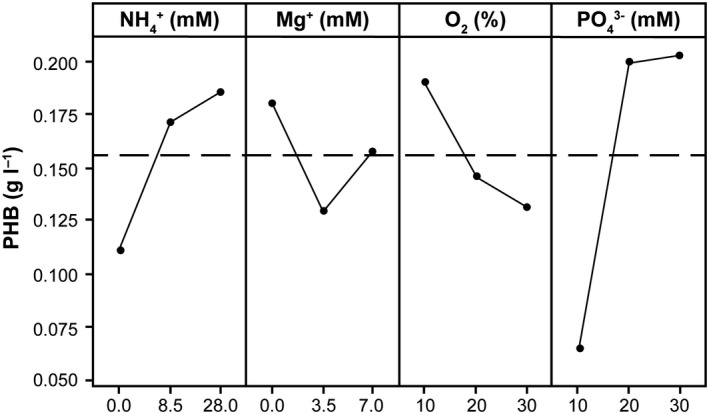
Effect of the variables on the production of PHB by *Caulobacter segnis* DSM 29236 (main effects). These variables were tested in a microreactor with medium MM3 and 3 ml of work volume (see Experimental procedures). Results were analysed by Taguchi method considering four factors: nitrogen source (NH_4_+ from (NH_4_)_2_ SO_4_), magnesium (Mg^2+^ from MgSO_4_), phosphates (${\text{PO}}_{4}^{3-}$ from buffer) and oxygen saturation and with PHB titre as the response.

Therefore, the process was further optimized in microreactor assays with 24 wells of 3 ml reaction volume. The temperature, pH and dissolved oxygen concentration were monitored and controlled via sensors in each well. A factorial design of experiments based on the orthogonal matrix method, whereby representative results can be obtained using some chosen variables, fixed at different levels, with the minimum possible number of experiments, was used. An orthogonal matrix L9 (3^4^) design that allows to study four factors or variables at three levels was chosen (Khosravi‐Darani *et al*., 2004; Wojtusik *et al*., 2015). This was intended to optimize both PHB production and lactose consumption starting with the simplest medium, MM3, the medium for which the highest yields had been obtained considering that the production of PHB is directly related to the composition of the medium and the oxygen saturation (Yang *et al*., 2010). Therefore, it was decided to add nitrogen source [NH_4_^+^ from (NH_4_)_2_SO_4_], magnesium (Mg^2+^ from MgSO_4_) and phosphates (${\text{PO}}_{4}^{3-}$ from buffer) to the medium and also test variations in oxygen saturation (Table 4). Experiments were designed accordingly to the L9 orthogonal array whereby nine experiments in duplicates were run. The amount of PHA produced by *C. segnis* DSM 29236 was not increased considerably, and lactose consumption rates were not entirely satisfactory using medium MM3 at these conditions (Table 4, exp. 1–9). However, results of the assays were analysed by Taguchi method taking PHB titre as a response since this is an intracellular product intrinsically related with cell growth. Components of the medium such as nitrogen, magnesium or phosphate play an essential role in the balance between growth and PHA accumulated by the strain (Bozorg *et al*., 2015). The effect of the variables selected in experimental design is shown in Fig. 3. In this case, the production of PHB tends to decrease when less amount of nitrogen source is added in the culture medium. According to literature, the limitation in nitrogen source increases the accumulation of PHA, but the accumulation of PHB remains around 10% practically in all trials. Therefore, the increase in the production of PHB is a consequence of an increase in cell growth due to the greater availability of nitrogen source in the culture medium. On the other hand, the effect of magnesium salt addition is not determinant. In terms of oxygen saturation and as expected, the production of PHB increases when there is oxygen limitation in the culture; therefore, it is important to control this variable using this strain in the same way as described for other PHA‐producing strains (Kshirsagar *et al*., 2012). Regarding the source of phosphate, there are also previous studies that show how the limitation in phosphate source improves the yields of PHA production, especially for cultures with high cell density (Lee *et al*., 2000). However, under the conditions tested, it is observed that at concentrations below 20 mM of phosphate the production of PHB decreases considerably; thus, this is a fundamental variable for the growth of the strain and the production of PHB.

**Table 4:** L9 matrix of the experimental design for the optimization of the PHA production using the simplest culture medium (MM3) and the microreactor (duplicate results)

Run	Variables (Factors)				Results			
	${\text{NH}}_{4}^{+}$ (mM)	Mg^2+^ (mM)	O_2_ (%)	${\text{PO}}_{4}^{3-}$ (mM)	OD_Final_	PHB (% wt)	PHB (g l^−1^)	Lactose (g l^−1^)
1	0.0 (1)	0.0 (1)	10 (1)	10 (1)	2.63 ± 0.03	11.1 ± 0.3	0.078 ± 0.003	1.56 ± 0.02
2	0.0 (1)	3.5 (2)	20 (2)	20 (2)	2.79 ± 0.02	9.8 ± 0.5	0.118 ± 0.052	1.28 ± 0.15
	0.0 (1)	7.0 (3)	30 (3)	30 (3)	2.96 ± 0.05	10.1 ± 0.2	0.135 ± 0.038	1.37 ± 0.05
4	8.5 (2)	0.0 (1)	10 (1)	30 (3)	4.89 ± 0.11	8.7 ± 0.4	0.233 ± 0.011	1.82 ± 0.17
5	8.5 (2)	3.5 (2)	30 (3)	10 (1)	4.69 ± 0.09	b.l.d	0.029 ± 0.016	1.90 ± 0.22
6	8.5 (2)	7.0 (3)	20 (2)	20 (2)	5.99 ± 0.22	8.7 ± 0.7	0.251 ± 0.038	1.70 ± 0.13
7	28 (3)	0.0 (1)	30 (3)	20 (2)	5.20 ± 0.07	8.3 ± 0.8	0.229 ± 0.052	n.d.
8	28 (3)	3.5 (2)	10 (1)	30 (3)	5.84 ± 0.04	8.7 ± 0.7	0.240 ± 0.054	2.14 ± 0.25
9	28 (3)	7.0 (3)	20 (2)	10 (1)	5.35 ± 0.13	b.l.d	0.087 ± 0.055	1.69 ± 0.08
Additional assays								
10	0.0	0.0	20.0	25.0	2.74 ± 0.03	11.9 ± 0.6	0.124 ± 0.032	1.22 ± 0.19
11	0.0	0.0	20.0	25.0	4.13 ± 0.12	b.l.d	0.080 ± 0.025	n.d.
12	15.0	0.0	20.0	25.0	6.23 ± 0.09	7.4 ± 0.3	0.222 ± 0.043	n.d.

b.l.d., below limit of detection.

Further trials were included: (i) a control experiment with the same conditions tested (MM3 unchanged); (ii) with added vitamins; and (iii) with an intermediate nitrogen concentration (Table 4, exp. 10–12). The results show that neither the addition of vitamins nor increasing the source of nitrogen improves the production of PHA with respect to the control conditions. Subsequent optimization assays were performed in other culture media to try to increase production yields considering the results mentioned above, particularly the C/N ratio influence.

Previous studies of fermentation with whey have shown that the C/N ratio is an important factor for the production of PHA. In some cases, it was found that PHB production was optimal for a C/N ratio of 50 with a consortium of microorganisms (Bosco and Chiampo, 2010), whereas with a strain of *B. thuringensis*, capable of producing appreciable amounts of PHB from whey, an optimal C/N ratio of 8 for PHA accumulation was found (Srividya, 2011; Gowda and Shivakumar, 2014). For this study, *C. segnis* DSM 29236 experiments were performed by modifying the inorganic nitrogen, phosphates and the amount of whey in the culture media: MM2, MM3, MM6 and MM7. The test conditions corresponding to the most relevant results are shown in Table 5. The C/N ratio of whole whey has a value of about 24 taking into account a typical elemental analysis; this is an intermediate value considering the C/N ratios mentioned above and it is interesting to avoid pretreatments such as ultrafiltration or protein precipitation, in order to simplify the process.

**Table 5:** Optimization of production of PHA with *Caulobacter segnis* DSM 29236‐modifying culture media MM2, MM3, MM6 and MM7 (triplicate results)

Medium	Modification	OD_Final_	PHB (% wt)	PHB (g l^−1^)	% Lactose Consumed
MM2a	0.1 g l^−1^ (NH_4_)_2_SO_4_, 8 g l^−1^ lactose	2.99 ± 0.07	5.9 ± 1.2	0.14 ± 0.01	70.8 ± 2.5
MM2b	0.1 g l^−1^ (NH_4_)_2_SO_4_, 16 g l^−1^ lactose	5.51 ± 0.06	8.5 ± 3.1	0.45 ± 0.05	63.4 ± 3.4
MM3a	0.1 g l^−1^ (NH_4_)_2_SO_4_, 8 g l^−1^ lactose	5.89 ± 0.11	31.5 ± 5.2	1.50 ± 0.08	74.5 ± 5.8
MM3b	0.2 g l^−1^ (NH_4_)_2_SO_4_, 8 g l^−1^ lactose	5.52 ± 0.08	23.1 ± 4.6	1.04 ± 0.22	66.5 ± 6.2
MM3c	Without (NH_4_)_2_SO_4_, 8 g l^−1^ lactose	4.05 ± 0.03	22.4 ± 2.8	0.78 ± 0.11	65.0 ± 4.6
MM6a	3.0 g l^−1^ (NH_4_)_2_HPO_4_, 6.67 g l^−1^ KH_2_PO_4_	2.41 ± 0.05	7.4 ± 1.5	0.15 ± 0.03	23.6 ± 5.7
MM6b	3.0 g l^−1^ (NH_4_)_2_HPO_4_, without KH_2_PO_4_	3.83 ± 0.14	12.2 ± 2.6	0.38 ± 0.02	36.7 ± 6.9
MM6c	1.0 g l^−1^ (NH_4_)_2_HPO_4_, 6.67 g l^−1^ KH_2_PO_4_	3.31 ± 0.07	9.2 ± 2.4	0.25 ± 0.01	42.6 ± 5.5
MM6d	4.0 g l^−1^ (NH_4_)_2_HPO_4_, 6.67 g l^−1^ KH_2_PO_4_	2.49 ± 0.05	7.4 ± 1.7	0.15 ± 0.04	32.3 ± 2.3
MM7a	16 g l^−1^ lactose	5.02 ± 0.10	7.7 ± 2.2	0.43 ± 0.09	52.7 ± 4.2
MM7b	8 g l^−1^ lactose	2.69 ± 0.02	8.8 ± 1.2	0.20 ± 0.07	80.9 ± 3.3

For the media MM2 and MM3, combinations were made using half the amount of whey, twice the original amount of nitrogen and the original amounts thereof maintaining nitrogen limitations and a C/N ratio of around 20. For the MM6 medium, the amounts of phosphate and nitrogen were modified maintaining a C/N ratio around 7 except for the MM6c medium which is 14. The MM7 medium is the medium richest in nitrogen, and only the amount of whey added is modified to observe the effects on lactose consumption. As Table 5 shows, the best results were obtained with medium MM3 with the modification MM3a, in which 31.5% of PHB was accumulated reaching 1.50 g l^−1^ of PHB. Lactose consumption increased considerably compared to previous trials. These conditions favour PHA accumulation as well as lactose consumption and cell growth. On the other hand, these results seem to indicate that a high concentration of phosphate and/or citric acid is detrimental to lactose consumption.

### Comparison of PHA production by *C. segnis* DSM 29236 and related *Caulobacter* strains from lactose contained in whey

**Figure 4:**
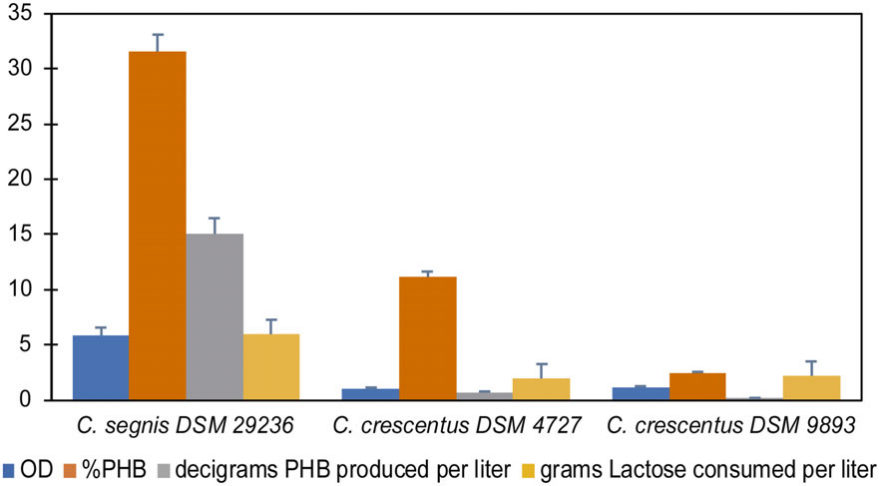
Two strains of the related species *C. crescentus* were chosen from the literature to test the highest PHA production capacity of *C. segnis* DSM 29236. The optimum operating conditions (see Experimental procedures) were tested using the mineral culture medium MM3a supplemented with whey as substrate.

*Caulobacter* are Gram‐negative bacteria with shapes that vary from rods, to fusiform, or vibrioid with asymmetric cell division to minimize competition for resources (Abraham *et al*., 1999). *Caulobacter segnis* is closely related to *Caulobacter crescentus*, which is an aquatic Gram‐negative alphaproteobacterium, and some strains were already described as PHA producers (Curtis and Brun, 2010; Patel *et al*., 2015). Two strains of the related species *C. crescentus* were chosen from the literature to test the highest PHA production capacity of *C. segnis* DSM 29236: *C. crescents* DSM 4727 and DSM 9893 (Qi and Rehm, 2001; Buckley, 2013). The optimum operating conditions were tested using the mineral culture medium MM3a supplemented with whey as carbon source to produce PHA from lactose. Batch incubation was performed in a flask at 30ᵒC and 180 rpm. The pH was set at 7, but it changed freely during the course of fermentation. Figure 4 shows the results obtained from incubations at 48 h. Strain *C. segnis* DSM 29236 was superior to *C. crescentus* strains, confirming that DSM 29236 is a good previously unknown PHA producer. In addition, to date, studies on PHA production with strains of the genus *Caulobacter* have been performed using glucose as carbon source, but not lactose. As mentioned above, there are few bacteria capable of using lactose directly as a carbon source. Some species of bacteria such as *B. megaterium* and *Methylobacterium* sp*. *ZP24 are able to use whole whey to produce PHB with biopolymer accumulation between 20 and 40% of CDW (Table 6) and even recombinant strains such as *E. coli* CGSC 4401 capable of producing 96.2 g l^−1^ of PHB and accumulating up to 80% of CDW in fed‐batch conditions (Ahn *et al*., 2000). Results obtained with this strain are quite similar to those obtained so far with previously reported wild‐type strains; therefore, it is a promising candidate for evaluating its PHB production performance in bioreactor under fed‐batch conditions.

**Table 6:** Production of PHA from whey in shaking flask and bioreactor: overview of literature data

Microorganism	CDW (g l^−1^)	PHA (g l^−1^)	PHA (g l^−1^ h^−1^)	Type of polymer	Device	References
*Azohydromonas lata*	9.20	1.66	0.02	PHBV	Flask	Baei *et al*. (2010)
	1.51	1.28	0.11	PHB	Flask	Berwig *et al*. (2016)
	1.67	0.16	0.01	PHB	Bioreactor	Berwig *et al*. (2016)
*Bacillus megaterium* CCM 2037	2.82	1.05	0.02	PHB	Flask	Obruca *et al*. (2011)
*Caulobacter segnis*	4.76	1.50	0.03	PHB	Flask	This study
	25.0	9.25	0.19	PHB	Bioreactor	This study
*Haloferax mediterranei*	11.0	5.50	0.05	PHB	Bioreactor	Koller *et al*. (2007)
*Halomonas halophila*	8.5	3.26	0.08	PHB	Flask	Kucera *et al*. (2018)
*Hydrogenophaga pseudoflava*	2.03	0.60	0.01	PHBV	Flask	Povolo *et al*. (2013)
	6.75	2.7	0.05	PHBV	Bioreactor	Koller *et al*. (2007)
*Methylobacterium* sp. ZP24	9.90	5.90	0.12	PHB	Flask	Yellore and Desai (1998)
	–	3.90	0.06	PHB	Bioreactor	Nath *et al*. (2008)
*Pseudomonas hydrogenovora*	10.8	1.30	0.03	PHB	Bioreactor	Koller *et al*. (2007)
	11.7	1.44	0.05	PHB	Bioreactor	Koller *et al*. (2008)
	10.6	1.27	0.04	PHBV	Bioreactor	Koller *et al*. (2008)
*Thermus thermophilus* HB8	1.43	0.50	0.01	PHA	Flask	Pantazaki *et al*. (2009)

PHA, containing C5, C7, C9 and C11 hydroxyalkanoic acids; PHBV, poly(3‐hydroxybutyrate‐co‐3‐hydroxyvalerate) copolymer.

### Validation of the production of PHB in fed‐batch cultures with *Caulobacter segnis* DSM 29236

**Figure 5:**
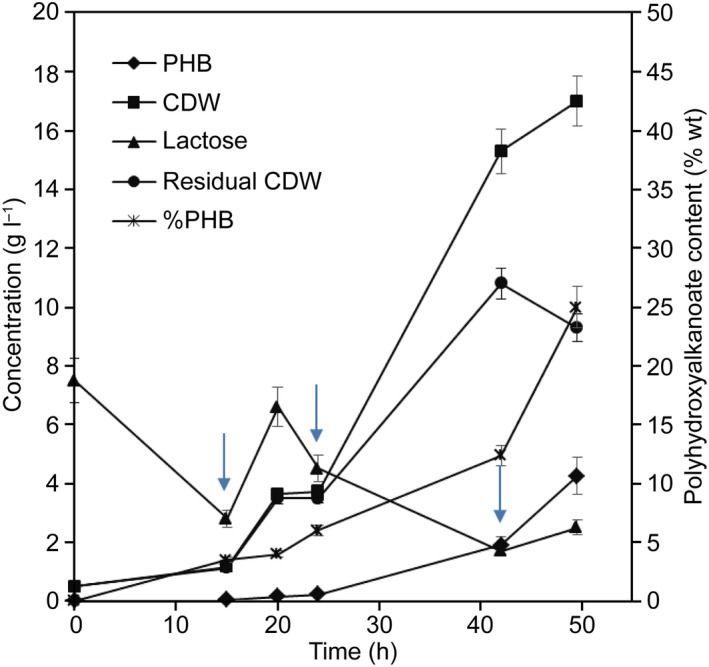
Growth and PHB production by *Caulobacter segnis* DSM 29236 for the fed‐batch fermentation with restricted medium MM3‐modified. Fermentation was done in a 1.5 l bioreactor with 0.5 l work volume and diluted whey (see Experimental procedures). Additions of whey have been made during fermentation at 15, 24 and 40 h as the arrows indicate.

Fed‐batch cultures have been used to evaluate the behaviour of the strain during PHA production and to optimize the fermentation to try to achieve high cell density cultures. As mentioned before, PHA is an intracellular product, so it is very important to obtain high cell density cultures as a step prior to the industrial scale‐up especially using waste streams as substrates (Rodriguez‐Perez *et al*., 2018). A first fermentation was performed using the MM3 medium supplemented with magnesium sulfate and ammonium sulfate to ensure the nutrients needed by the strain during the first phase of the fermentation. Again, the MM3 medium was used as the culture medium with which the strain accumulated the greatest amount of PHB in the previous assays. The results of the experiment are shown in Fig. 5. During the experiment, oxygen saturation was decreasing from the beginning of the fermentation until it remained at 30%. After 15 h of fermentation, the oxygen saturation started to rise gradually, and the lactose concentration was 2.5 g l^−1^, so an amount of whey equivalent to 20% (100 ml) of the batch volume was added. Then, the oxygen saturation began to drop again and remained at 30% while the cell density of the culture continued increasing. At 24 h, a second addition of whey of around 40% of the initial batch volume (200 ml) was made and the culture continued to grow and consume lactose. At 40 h, the culture was very viscous and the pH adjustment with ammonium was changed to sodium hydroxide 5 N to maintain nitrogen limitation during the last fermentation stage. A final addition of whey equivalent to 40% of the initial batch was made, and the fermentation was finished after 50 h. Under these conditions, the strain achieved an accumulation of PHB of 25% of its CDW, 4.3 g l^−1^ of PHB was obtained and the strain consumed almost all the lactose. The production of PHB obtained with this experiment is about three times higher than the value obtained in flask. In addition, the amounts of whey added during fermentation have diluted the culture considerably, but it has shown that this substrate at high concentration has no inhibitory effects on the strain. However, inhibitory effects are observed when the strain is not adapted to the culture medium and high concentrations of the whey are added from the beginning of the fermentation.

**Figure 6:**
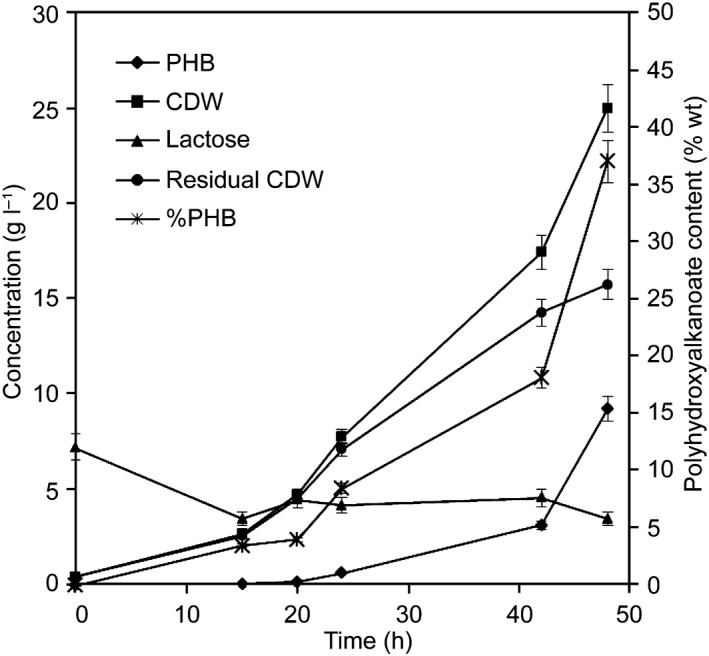
Growth and PHB production by *Caulobacter segnis* DSM 29236 for the fed‐batch fermentation with continuous addition of concentrated whey to maintain the culture with a concentration around 5 g l^−1^ of lactose. Fermentation was done in a 1.5‐l bioreactor with 0.5 l work volume and diluted whey in MR medium (see Experimental procedures).

A second fed‐batch fermentation experiment was performed to evaluate the production of PHB in a less limited culture medium with more nitrogen and phosphorus sources and to test the continuous addition of a concentrated solution of whey. For this purpose, the MR medium described by Lee *et al*. (2000) and a fivefold concentrated whey solution were used to achieve a lactose concentration of about 200 g l^−1^. Figure 6 shows the results of the experiment. As in the previous experiment, the oxygen saturation of the culture started to fall since the beginning of the fermentation and at 15 h the feed of concentrated substrate was activated to maintain the concentration of lactose in the culture at around 5 g l^−1^ during the fermentation. The oxygen saturation of the culture was maintained at 30% during the whole fermentation, and the cell density was gradually increasing during the experiment. In a similar way to the previous experiment, the pH control with ammonium was changed to NaOH 5 N at 40 h of fermentation and the culture showed less viscosity. Finally, an accumulation and PHB concentration of 37% and 9.25 g l^−1^ are obtained, respectively, and the strain has consumed lactose efficiently.

These first fed‐batch fermentation experiments constitute the first steps to the scale‐up for the production process of PHB with strain *C. segnis* DSM 29236. The strain has reached PHB concentrations around 10 g l^−1^, almost seven times more than the results obtained in the flask cultures. The selection of convenient batch medium and adjusted feeding policy has allowed us to achieve the highest reported PHB concentration for a wild‐type microorganism (Table 6). Therefore, the production of PHB has been validated with a wild strain not previously described as producing PHB and it has been demonstrated its suitability as industrial strain.

Probably, the advances in systems and synthetic biology will enable the design and construction of PHA‐hyperproducing strains (Wang *et al*., 2014; Chen and Jiang, 2017). For example, cells could be induced for flocculation precipitation followed by induced cell lysis after completing PHA production, and fermentation could be conducted continuously under unsterile conditions using seawater so that PHA production costs would make them competitive with petroleum‐based plastics (Martinez *et al*., 2011; Tan *et al*., 2011).

While synthetic engineered strains are still being developed for industrial PHA production, a new wild‐type strain capable of producing PHA from lactose and whey has been identified by an *in silico* prospecting procedure and avoiding the use of genetic engineering techniques to obtain GMOs. As PHA has been successfully validated as in food packaging applications (Bucci *et al*., 2005), the transformation of whey into PHB is also a relevant example of circular economy for the food value chain. From a regulatory standpoint, whey may be considered a preferred raw material in relation to other industrial by‐product streams in terms of food safety compliance, particularly to derive materials for food storage. This means a step forward for bioplastics production and progress in the development of safer and more efficient sustainable technologies.

## Conclusions

The purpose of this work was to find new bacteria capable of producing PHA from whey at good yields. *In silico* selection of strains and the Nile red method have demonstrated the ability of some biosafety level‐1 bacteria to produce PHA from whey and permeate. Following a funnel strategy, starting from collection strains, the production process of PHA was optimized with strain *C. segnis* DSM 29236, which proved a good candidate for PHB production from whey. It is a Gram‐negative rod‐ or vibrioid‐shaped or fusiform bacterium, which has never been related to PHB production previously. The strain produced 1.5 g l^−1^ of PHB (31.5% of CDW) in discontinuous culture under optimized conditions found by modifying the amounts of nitrogen and carbon source of the culture media to achieve the appropriate production conditions. In addition, fed‐batch fermentation assays have been carried out with *C. segnis* DSM 29236 reaching up to 9.25 g l^−1^ of PHB with a 37% accumulation of its CDW and with potential for improvement. This is the highest concentration reported to date, to our best knowledge, for a wild‐type microorganism capable to directly hydrolyse and transform lactose from whey into PHB. Thus, this study is a promising step in the design of a sustainable and safe biotechnological process to produce biodegradable bioplastics using industrial waste as raw material and wild‐type strain as PHA producer.

## Experimental procedures

### Bacterial strains, media and growth conditions

The bacterial strains employed in this study are listed in Table 2. The selected strains were purchased from the Spanish (CECT), German (DSMZ) and British (NCIMB) Type Culture Collections (Table 2). After receiving the strains, they were recovered in the media recommended by the supplier and stored in 20% glycerol for long‐term preservation.

Cells were initially cultured in an undefined rich medium (Luria‐Bertani, LB). The strains were precultured in mineral medium 1 (MM1) [0.5 g Na_2_SO_4_ l^−1^, 7.5 g KH_2_PO_4_ l^−1^, 10 g (NH_4_)_2_HPO_4_ l^−1^ and 25 ml per litre of trace elements solution: 0.5 g MgSO_4_·7H_2_O l^−1^, 2 mg H_3_BO_3_ l^−1^, 1 CuSO_4_·5H_2_O mg l^−1^, 1.3 mg Na_2_MoO_4_·2H_2_O l^−1^, mg ZnSO_4_·7H_2_O 11 l^−1^, 2 mg CoCl_2_·6H_2_O l^−1^, 7 mg MnSO_4_·H_2_O l^−1^, 35 mg FeSO_4_·7H_2_O l^−1^, 2.5 g KH_2_PO_4_ l^−1^, 50 mg CaCl_2_·2H_2_O l^−1^, 0.5 g citric acid l^−1^, 0.35 mg KI l^−1^, 0.5 g Al_2_(SO_4_)_3_ l^−1^], with lactose as sole carbon source in order to adapt them to the presence of sugar (Young *et al*., 1994). Solid media were supplemented with 1.5% (w/v) agar.

Different culture media, including diluted whey, were used to optimize PHA production with *Caulobacter segnis* DSM 29236: Mineral Medium 2 (MM2) [1.2 g Na_2_HPO_4_ l^−1^, 0.33 g KH_2_PO_4_ l^−1^, 0.11 g NH_4_Cl l^−1^, 0.1 g MgSO_4_·7H_2_O l^−1^, 0.04 g CaCl_2_ l^−1^. Vitamins 1000x: 20 mg biotin l^−1^, 20 mg folic acid l^−1^, 10 mg pyridoxine·HCl l^−1^, 50 mg thiamine·HCl·2H_2_O l^−1^, 50 mg riboflavin l^−1^, 50 mg niacin l^−1^, 50 mg D‐pantothenate calcium l^−1^, 50 mg vitamin B12 l^−1^, 50 mg p‐aminobenzoic acid l^−1^. Trace elements 1000x: 1.5 g nitrilotriacetic acid l^−1^, 0.18 g ZnSO_4_·7H_2_O l^−1^, 3 g MgSO_4_·7H_2_O l^−1^, 0.01 g CuSO_4_·5H_2_O l^−1^, 0.5 g MnCl_2_·4H_2_O l^−1^, 0.02 g KAl(SO_4_)_3_·12H_2_O l^−1^, 1 g NaCl l^−1^, 0.01 g H_3_BO_3_ l^−1^, 0.1 g FeSO_4_·7H_2_O l^−1^, 0.01 g Na_2_MoO_4_ l^−1^, 0.18 g CoSO_4_·7H_2_O l^−1^, 0.025 g NiCl_2_·6H_2_O l^−1^, 0.3 g NaSeO_3_·5H_2_O l^−1^] (López Barragán *et al*., 2004), Mineral Medium 3 (MM3) [2.5 g Na_2_HPO_4_ l^−1^, 1.0 g KH_2_PO_4_ l^−1^. Trace elements 400 × 5 M HCl: 10 g FeSO_4_·7H_2_0 l^−1^, 2 CaCl_2_·2H_2_O g l^−1^, 2.2 g ZnSO_4_·7H_2_O l^−1^, 0.5 g MnSO_4_·4H_2_O l^−1^, 1 g CuSO_4_·5H_2_O l^−1^, 0.1 g (NH_4_)_6_Mo_7_O_24_·4H_2_O l^−1^, 0.02 g Na_2_B_4_O_7_·10H_2_O l^−1^, 8 mg NiCl_2_·6H_2_O l^−1^, 2.4 mg CoCl_2_·6H_2_O l^−1^] (Yellore and Desai, 1998), Mineral Medium 4 (MM4) [whey supplemented with FeCl_3_ and vitamins] (Povolo and Casella, 2003), Mineral Medium 5 (MM5) [0.5 g NHCO_3_ l^−1^, 2.9 g Na_2_HPO_4_ l^−1^, 2.3 g KH_2_PO_4_ l^−1^, 0.5 g MgSO_4_·7H_2_O l^−1^, 2 g (NH_4_)_2_SO_4_ l^−1^, 0.01 g CaCl_2_·2H_2_O l^−1^, 0.05 g NH_4_Fe(III) citrate l^−1^ and 5 ml per litre of SL6 solution: 100 mg ZnSO_4_·7H_2_0 l^−1^, 300 mg H_3_BO_3_ l^−1^, 200 mg CoCl_2_·6H_2_O l^−1^, 6 mg CuSO_4_ l^−1^, 20 mg NiCl_2_·6H_2_O l^−1^, 30 mg Na_2_MoO_4_·12H_2_O l^−1^, 25 mg MnCl_2_·2H_2_O l^−1^] (Koller *et al*., 2007), Mineral Medium 6 (MM6) [6.67 g KH_2_PO_4_ l^−1^, 4 g (NH_4_)_2_HPO_4_ l^−1^, 0.8 g MgSO_4_·7H_2_O l^−1^, 0.8 g citric acid l^−1^ and 5 ml per litre of solution trace elements (5 M HCl): 10 g FeSO_4_·7H_2_0 l^−1^, 2 g CaCl_2_·2H_2_O l^−1^, 2.2 g ZnSO_4_·7H_2_O l^−1^, 0.5 g MnSO_4_·4H_2_O l^−1^, 1 g CuSO_4_·5H_2_O l^−1^, 0.1 g (NH_4_)_6_Mo_7_O_24_·4H_2_O l^−1^, 0.02 g Na_2_B_4_O_7_·10H_2_O l^−1^] (Ahn *et al*., 2000) and Mineral Medium 7 (MM7) [5 g (NH_4_)_2_SO_4_ l^−1^, 2.5 g Na_2_HPO_4_ l^−1^, 2.5 g KH_2_PO_4_ l^−1^, 0.2 g MgSO_4_ l^−1^ and 0.01 g MnSO_4_ l^−1^] (Obruca *et al*., 2011). The pH value was set to 7 in all cases, and then, whey was added to achieve an initial lactose concentration of 12 g l^−1^. Bacteria were incubated in flasks with stirring, at 30°C and 180 rpm. Growth was measured by absorbance at 600 nm in a spectrophotometer or by cell dry weight (CDW) determination of freeze‐dried samples. Optimization assays were carried out in the microreactor Applikon μ‐24, with 3 ml of reaction volume, at 30°C and stirring at 500 rpm. pH was left to develop without restraint.

### Fed‐batch cultures

Seed cultures for the fed‐batch cultures were prepared in flasks containing LB medium by incubating in a rotary shaker overnight at 30°C and 150 rpm. The experiments were performed in a 1.5‐l Applikon^®^ reactor with a working volume of 0.5 litre at 30°C. pH was controlled at 7.0 by the addition of 25% v/v ammonia. The oxygen saturation was controlled at 30% by controlling the agitation speed and at 1 vvm of air flow rate. Foaming was controlled by adding Antifoam 204 (Sigma‐Aldrich, St. Louis, MO, USA) when necessary. Two sets of fermentations were carried out with *Caulobacter segnis* strain: (i) fermentation with MM3‐modified medium supplemented with 0.5 g l^−1^ MgSO_4_ and 0.1 g l^−1^ (NH_4_)_2_SO_4_ with subsequent feedings of whey and (ii) fermentation with MR medium (Lee *et al*., 2000) with the initial KH_2_PO_4_ concentration set at 4 g l^−1^ and continuous feeding of concentrated whey with around 200 g l^−1^ of lactose by using dry whey diluted in water (×5). Detailed feeding strategies are described in the results section.

### Analytical methods

The content of lactose, glucose and lactic acid was determined by HPLC liquid chromatography (2695 HPLC with a Refractive Index Detector 2414; Waters, Cerndanyola del Vallés, Spain) using a Rezex ROA Organic Acid Column, with H_2_SO_4_ at 2.5 mM and 0.5 ml min^−1^ flow. Additional analysis of lactose was performed with the Colorimetric Kit Megazyme K‐LACGAR Lactose and D‐Galactose (Rapid, BIOCON‐Española, Barcelona, Spain). Protein was measured with a Thermo Fisher Commercial Kit (BCA Protein Assay Kit; Pierce, Fisher Scientific‐Spain, Madrid, Spain) using the *BCA* method. Fat was determined by Soxhlet extraction of dried whey with chloroform. The ash content was determined in a high‐temperature muffle furnace, Nabertherm GmbH. A CE Instruments CHN1100 elemental analyser was used to perform an elemental analysis of whey by atomic spectrometry of C, N, H and S. Oxygen content is estimated by difference. Data are shown in dry basis.

Polyhydroxyalkanoate was determined by methanolysis and gas chromatography of lyophilized culture samples. For this purpose, after 48 h of incubation, biomass was collected from 50 ml cultures by centrifugation and lyophilized. Methanolysis reaction was performed weighing 5–7 mg of dry cells, using H_2_SO_4_ in 15% (v/v) in methanol and chloroform, and incubating for 4 h at 100°C to transform the PHA into hydroxy methyl esters. Methyl benzoate was used as an internal standard. The resulting methyl esters were collected in the chloroform phase and analysed by gas chromatography (Lageveen *et al*., 1988). Different pure commercial PHA samples were used as bioplastic standard: PHB poly(3‐hydroxybutyrate), PHBV poly(3‐hydrobutyrate‐co‐3‐hydroxyvalerate) and PHO poly(3‐hydroxyhexanoate‐co‐3‐hydrooctanoate). The equipment used was an Agilent 6890N gas chromatograph with flame ionization detector (FID) and HP5‐MS column.

Polyhydroxyalkanoate quantification for microreactor assays was performed with slight modifications (Cruz *et al*., 2016). Briefly, 2–3 mg of dry cells was obtained from 3 ml of a culture of *Caulobacter segnis* with MM3 medium considering that a concentration of 0.7 g l^−1^ of CDW was reached (Fig. 2). Samples were weighed at an analytical balance with a precision of 0.1 mg, imposing an error of 4% of total biomass. Therefore, PHB percentages below 4% are rejected. This biomass amount corresponds to half of the sample used in the method described above; therefore, in this case methanolysis reagents were used at half volume for 3.5 h at 100°C.

### Whey preparation

In order to use the whey as a basis for bacterial cultures, it must first be sterilized. Two methods were followed: filtering or heating in an autoclave. In the former, the whey was passed through several filters (25, 8, 1, 0.45 and 0.22 μm) to remove suspended particles and microorganisms; in the latter, sterilization was performed at 121°C at 1 atm in the autoclave.

### Screening with Nile red

Microorganisms were inoculated in a non‐PHA production medium (LB), in a nitrogen‐limited PHA production medium, 0.1 N M63 (de Eugenio *et al*., 2010) with 20 g l^−1^ glucose or 12 g l^−1^ lactose and in a medium prepared by diluting whey or permeate to a lactose concentration of 12 g l^−1^. The fluorescence spectrometer used was Hitachi F7000 (Hitachi Europe, Krefeld, Germany) with λ_excitation_ = 545 nm and λ_emission_ = 598 nm. The protocol was adapted from a simple and highly sensitive method to detect PHA from growing colonies (Spiekermann *et al*., 1999; Cruz *et al*., 2016). Assays were performed in multiwell plates with volume of 200 μl and incubating at 30°C and 500 rpm for 24 h. The initial OD_600_ of the cultures was set at 0.3, and the OD_600_ at 24 h of each strain was adjusted to the lowest OD_600_ obtained to normalized cell densities for RFU measurements. Then, to each well, 2 μl of a solution of 0.1 g Nile red per litre of DMSO was added and fluorescence was assessed in multiwell black plates after 15 min of incubation and compared with the reading taken without the dye.

### Statistical *design* of experiments

A design of experiment (DOE) technique was used to test the relative importance of medium components and environmental factors on PHA production. Results of the microreactor assays were obtained and analysed employing the Taguchi method, which is based on an orthogonal fractionated factorial design methodology. The application of this methodology results in a dramatic reduction in the number of experiments to be performed (Rao *et al*., 2008; Velasco *et al*., 2017).

### *In silico* analysis

For the identification of PHA‐producing microorganisms from lactose, the BLAST (Basic Local Alignment Search Tool) program was used. BLAST finds regions of similarity between biological sequences. The program compares nucleotide or protein sequences to sequence databases and calculates the statistical significance (Mount, 2007).

## Conflict of interest

None declared.

## Supporting information


**Fig. S1**. *In silico* prospecting for PHA producers with beta‐galactosidase activity.
**Fig. S2**. Chromatogram of PHA sample.
