# Supplementary material for: In silico prospection of microorganisms to produce polyhydroxyalkanoate from whey: Caulobacter segnis DSM 29236 as a suitable industrial strain
Source: Microb Biotechnol. 2019 Jan 31;12(3):487–501. doi: 10.1111/1751-7915.13371 (PMC6465232; doi:10.1111/1751-7915.13371)
Supplement: Supplementary file 1 — Fig. S1. In silico prospecting for PHA producers with beta‐galactosidase activity. Fig. S2. Chromatogram of PHA sample. [file MBT2-12-487-s001.docx]

SUPPLEMENTARY

FIGURE S1 : ***In silico* prospecting for PHA producers with beta-galactosidase activity.** A list of bacteria with information related with recommended growth temperature (name color) and biosafety level (pink dots) was classified according to the different *in silico* screening protocols (see *Experimental procedures*). The central diagram shows the set of strains selected for further studies. (* indicates that previous studies define growth from lactic acid).


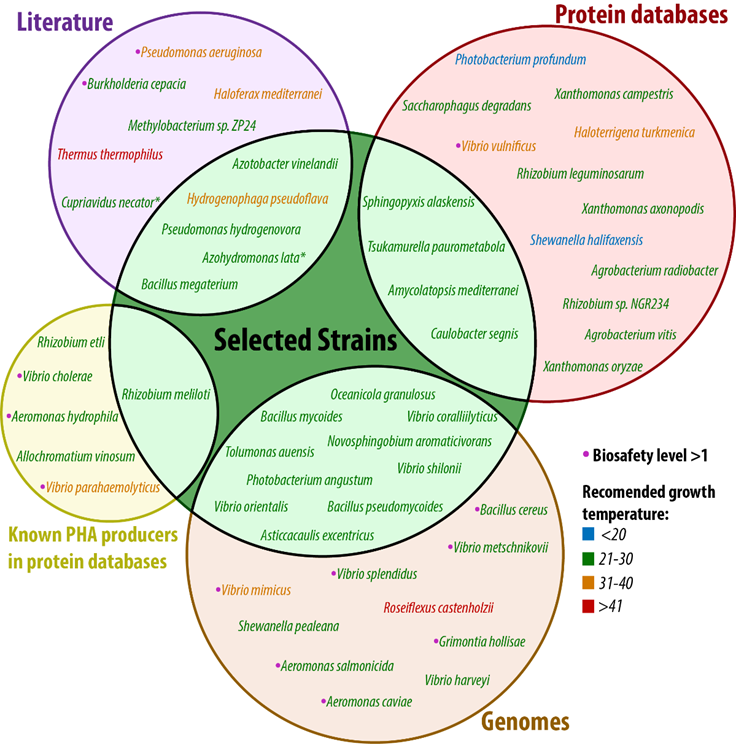


FIGURE S2: Chromatogram of PHA sample. After methanolysis of the sample, the analysis is performed by GC-FID (gas chromatography-flame ionization detector) showing internal standard (methyl benzoate) and PHB monomers as 3-hydroxymethylesters in the *Caulobacter segnis* DSM 29236 dry biomass (see *Experimental procedures*). *C. segnis* DSM 29236 is identified as a PHB producer.
